# Efficacy of ursodeoxycholic acid in metabolic dysfunction-associated steatotic liver disease: an umbrella review of meta-analyses on liver enzymes

**DOI:** 10.3389/fmed.2026.1771830

**Published:** 2026-02-13

**Authors:** Alsu R. Khurmatullina, Dmitrii N. Andreev, Igor V. Maev, Andrey V. Zaborovsky, Yury A. Kucheryavyy, Petr A. Beliy, Philipp S. Sokolov

**Affiliations:** 1 Department of Internal Disease Propaedeutics and Gastroenterology, Russian University of Medicine, Moscow, Russia; 2 Department of Pharmacology, Russian University of Medicine, Moscow, Russia; 3 Ilyinskaya Hospital, Krasnogorsk, Russia

**Keywords:** MASLD, meta-analysis, UDCA, umbrella review, ursodeoxycholic acid

## Abstract

**Objective:**

This umbrella review aimed to systematically synthesize and evaluate evidence from published meta-analyses regarding the efficacy of ursodeoxycholic acid in metabolic dysfunction–associated steatotic liver disease, focusing on its impact on liver-specific biochemical markers.

**Methods:**

Following Joanna Briggs Institute methodology and registered in PROSPERO (ID: CRD420251250211), a comprehensive search of MEDLINE, EMBASE, Cochrane Library, and Scopus (1985–2025) identified systematic reviews and meta-analyses evaluating UDCA therapy in MASLD. Methodological quality was appraised using AMSTAR-2, ROBIS, and GRADE frameworks. Data on hepatic biomarkers were extracted and synthesized using fixed- or random-effects models depending on heterogeneity (I^2^ statistic). Overlap among primary studies was assessed using the GROOVE tool, and meta-regression explored the influence of treatment duration on ALT dynamics.

**Results:**

Five meta-analyses (33 primary studies; 5,015 participants) were eligible. UDCA demonstrated consistent and statistically significant improvements in key markers of hepatocellular injury, including ALT (SMD = −0.36; 95% CI: −0.69 to −0.03) and AST (SMD = −0.16; 95% CI: −0.22 to −0.10), as well as cholestatic markers such as GGT (SMD = −0.40; 95% CI: −0.63 to −0.18) and ALP (SMD = −0.23; 95% CI: −0.31 to −0.14), total bilirubin decreased modestly (SMD = −0.08; 95% CI: −0.15 to −0.01), while albumin level remained unchanged. Meta-regression showed that longer treatment duration was significantly associated with greater ALT reduction (−0.04 SMD per 6 months; *p* = 0.034).

**Conclusion:**

UDCA demonstrates consistent hepatoprotective and cholestasis-modifying effects in MASLD. Longer treatment duration may enhance biochemical responses.

**Systematic review registration:**

CRD420251250211.

## 1 Introduction

Metabolic dysfunction–associated steatotic liver disease (MASLD), formerly termed non-alcoholic fatty liver disease (NAFLD), is one of the most prevalent chronic liver conditions worldwide, affecting up to 38% of adults and substantially contributing to global liver-related morbidity and mortality (1–4). MASLD is closely linked to metabolic comorbidities like insulin resistance, obesity, and type 2 diabetes. These conditions not only promote hepatic steatosis but also accelerate its progression to steatohepatitis, fibrosis, cirrhosis, and hepatocellular carcinoma, while also increasing the risk of cardiovascular disease (5, 6). Given the potential for disease progression, there is a clear need for pharmacotherapy that directly targets underlying hepatic inflammation to reduce the risk of long-term complications. Despite this pressing clinical need and the serious long-term consequences of MASLD, no universally approved pharmacological therapy currently exists to halt or reverse its progression (7). In this context, ursodeoxycholic acid (UDCA), a hydrophilic bile acid with established cytoprotective, anti-apoptotic, anti-inflammatory, and choleretic properties, has emerged as a potential therapeutic candidate capable of targeting several key pathophysiological mechanisms involved in MASLD (8, 9).

Evidence from clinical trials evaluating UDCA in MASLD has remained mixed. A meta-analysis of seven randomized controlled trials demonstrated that UDCA reduced alanine aminotransferase (ALT, AST, and GGT) levels compared with control therapy (SMD −0.18, 95% CI: −0.28 to −0.08), −0.18 (95% CI: −0.27 to −0.1), and −0.21 (95% CI: −0.31 to −0.11) (10). Importantly, beyond its potential hepatoprotective effects, emerging research has suggested that UDCA may exert favourable influences on cardiometabolic risk factors—such as lipid metabolism, glycaemic control, systemic inflammation, and markers of atherogenesis—which are integral to both the progression of MASLD and its extrahepatic complications (11–13). Subsequent meta-analyses, including broader patient populations and longer follow-up durations, have reported heterogeneous findings, thereby limiting the certainty of evidence regarding UDCA’s therapeutic role.

In MASLD, where histologic verification is not always feasible, biochemical markers serve as crucial surrogate endpoints for disease activity. Their reduction is a clinically significant treatment goal, as it correlates with improved hepatic histology and a lower risk of disease progression (14).

However, these outcomes have not been comprehensively synthesised across existing meta-analyses.

Given this gap, the present umbrella review aims to consolidate and critically appraise evidence from published meta-analyses on the efficacy of UDCA for MASLD, focusing on liver-specific biomarkers.

## 2 Materials and methods

### 2.1 Search strategy

We performed this umbrella review in accordance with the methodological standards of the Joanna Briggs Institute (15). This approach is particularly suitable when multiple systematic reviews address related research questions, as it enables consolidation of their findings, identification of concordant and discordant evidence, and detection of persisting knowledge gaps relevant to clinicians, policymakers, and researchers. Our methodology followed current best practices and was aligned with previously published umbrella reviews (16). The review protocol was prospectively registered in the PROSPERO database (registration ID: CRD420251250211). A comprehensive literature search was conducted following the PRISMA 2020 reporting guidelines for systematic reviews and meta-analyses (17). The completed PRISMA checklist is provided in the Supplementary Materials (Supplementary Table S1).

To ensure full coverage of the available evidence, we performed a systematic search across major electronic databases, including MEDLINE/PubMed, EMBASE, Cochrane Library, and Scopus. The search covered the period from January 1, 1985, to November 10, 2025. Eligible meta-analyses were those synthesizing original studies that evaluated the effects of UDCA on MASLD, MASLD-related hepatic outcomes and their associated biomarkers. Reviews without quantitative pooled analyses and those examining unrelated interventions or populations were excluded.

We developed predefined search strategies addressing hepatic domain. For MASLD-related hepatic outcomes, the following strategy was used in PUBMED: (“Fatty Liver” [MeSH] OR “Non-alcoholic Fatty Liver Disease” OR NAFLD OR MASLD OR NASH “metabolic dysfunction–associated steatotic liver disease”) AND (“Ursodeoxycholic Acid”[MeSH] OR UDCA OR ursodiol).

This search strategy was adapted as necessary for each database, which can be found in Supplementary Materials. Filters were applied to identify meta-analyses and systematic reviews.

### 2.2 Eligibility criteria and quality assessment

The methodological framework followed the PICO approach. The Population included adult patients diagnosed with MASLD or NAFLD or NASH. Only studies explicitly analyzing human subjects were eligible; research based on animal models or *in vitro* experiments was excluded. The Intervention of interest was treatment with UDCA. The Comparison group consisted of placebo, standard care without UDCA, or alternative active treatments. The Outcomes of interest encompassed hepatic biomarkers, along with reported effect sizes (standardized mean differences) and indicators of heterogeneity and methodological quality. The Study design was limited to systematic reviews that synthesized randomized or non-randomized controlled clinical trials. No language restrictions were applied. Two independent reviewers (A. R. K. and D. N. A.) screened the studies, while a third independent reviewer (Y. A. K.) solved the conflicts through discussion process.

Insufficient methodological rigor was defined *a priori* based on critical domains of the AMSTAR-2 tool. Systematic reviews were excluded if they failed to meet one or more of the following key methodological criteria: absence of a clearly defined PICO question; lack of a comprehensive and reproducible literature search strategy; absence of a risk-of-bias assessment of the included primary studies; unclear or inappropriate methods for data synthesis. Reviews failing in these critical domains were considered methodologically unreliable for inclusion in an umbrella review.

Two independent reviewers (F. S. S. and Y. A. K.) assessed the methodological quality of eligible systematic reviews and meta-analyses using a modified version of the AMSTAR-2 tool (A Measurement Tool to Assess Systematic Reviews) (18), while conflicts were solved by D. N. A. The AMSTAR-2 tool consists of 16 items evaluated as “yes” (criterion fully met), “no” (criterion unmet or insufficiently addressed), or “partial yes” (criterion partially satisfied).

### 2.3 Risk of bias evaluation

The risk of bias in randomized controlled trials included within the systematic reviews was appraised using the ROBIS instrument (Risk of Bias in Systematic Reviews) (19). The tool evaluates five domains: (1) Study eligibility criteria; (2) Identification and selection of studies; (3) Data collection and appraisal; (4) Synthesis and findings; and (5) Overall risk of bias. Each domain contains signaling questions guiding the assessment, with judgments categorized as “low risk,” “some concerns,” or “high risk.” Certainty of evidence for all evaluated associations was further assessed according to the updated GRADE framework (Grading of Recommendations Assessment, Development and Evaluation) (20).

Two reviewers (P. B. A. and A. Z. V.) independently performed the assessments. Disagreements were resolved through discussion wuth the third independent reviewer (F. S. S.). For visualization, ROBIS-derived risk-of-bias ratings across all domains and the overall assessment were depicted using traffic-light style plots.

### 2.4 Overlap of primary studies

The degree of overlap among primary studies included across the systematic reviews was quantified using the GROOVE tool (Graphical Representation of Overlap for OVErviews) (21). GROOVE generates an evidence matrix, calculates the number of unique and overlapping studies, and reports the Corrected Covered Area (CCA), which serves as a standardized summary measure of overlap. To evaluate whether this overlap resulted in disproportionate weighting of frequently included primary studies, a leave-one-out sensitivity analysis was performed. Meta-analyses containing the most commonly repeated randomized controlled trials were sequentially excluded, and pooled effect estimates for the primary outcomes were recalculated. The leave-one-out analysis demonstrated that exclusion of these overlapping studies did not materially alter the magnitude, direction, or statistical significance of the pooled effects. Overall effect estimates for key biochemical markers remained stable across all iterations, indicating that the observed benefits of UDCA were not driven by overrepresentation of repeatedly included primary studies despite the high degree of overlap.

### 2.5 Data extraction

Screening and data extraction were conducted by two independent reviewers (A. R. K. and D. N. A.) and any disputes were solved by I. V. M. The initial screening phase involved evaluating titles, abstracts, and keywords for relevance. If consensus was not reached or if abstracts lacked sufficient detail, the full text was retrieved for evaluation. A second screening phase was performed based on full-text assessment to ensure that all eligibility criteria were satisfied. Data extraction followed a standardized protocol.

From each systematic review, we recorded the publication year, number of included primary studies, target condition, UDCA treatment regimens, population characteristics, outcome definitions, the number of effective cases, and the statistical models applied. Additionally, we extracted data on hepatic endpoints (e.g., ALT, AST, and GGT).

Comparative effectiveness was extracted primarily as mean difference (MD), weighted mean difference (WMD), or standardized mean difference (SMD), depending on the reporting of the original meta-analyses.

### 2.6 Statistical analysis

Following data extraction, continuous outcomes reported in the original meta-analyses—such as liver markers levels—were harmonized and recalculated using mean difference–based effect measures (SMD), consistent with the reporting conventions of the included reviews. For these outcomes, negative values reflected improvement when lower levels were clinically favorable (e.g., ALT, AST), whereas positive values indicated improvement when higher levels were beneficial (e.g., albumin levels).

Given the anticipated heterogeneity across populations, interventions, and outcome definitions, a random-effects model was applied whenever *I*^2^ exceeded 50%. Between-study heterogeneity was assessed using the *I*^2^ statistic.

Potential publication bias was examined using funnel plot asymmetry and Egger’s regression test. All outcomes were reported with 95% confidence intervals, and statistical significance was defined as *p* < 0.05. Analyses were conducted using RevMan software (version 5.4.1; London, England).

## 3 Results

### 3.1 Study selection

**Figure 1:**
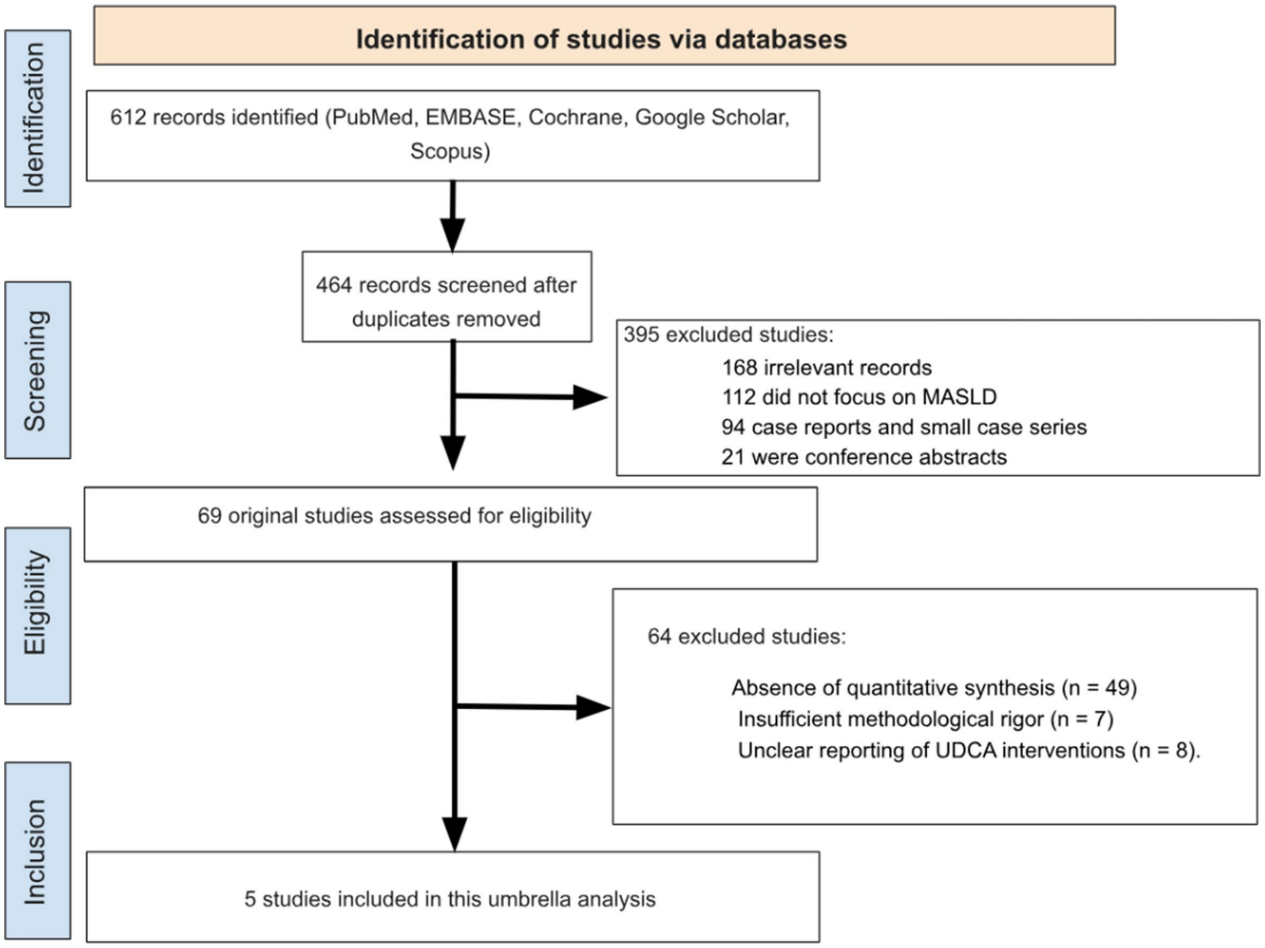
PRISMA (preferred reporting items for systematic reviews and meta-analyses) flowchart.

A total of 612 records were identified through searches of the electronic databases. After removal of 148 duplicates, 464 unique records were screened by title and abstract. Of these, 395 records were excluded because they did not investigate UDCA, did not focus on MASLD, or were unrelated to hepatic biomarkers. The full texts of the remaining 69 articles were retrieved for detailed evaluation. Following full-text review, 64 studies were excluded for the following reasons: absence of quantitative synthesis (*n* = 49), insufficient methodological rigor (n = 7), or unclear reporting of UDCA interventions (*n* = 8). Ultimately, 5 meta-analyses met all eligibility criteria and were included in the umbrella review (Figure 1), (9, 11, 22–24).

Table 1 provides an overview of the main features of the included systematic reviews and meta-analyses, including their primary outcomes, total sample sizes, comparator groups, risk-of-bias assessments, and overall quality appraisals.

**Table 1:** Characteristics of included studies.

Author, year	Types of included studies and number of studies, n	No. of included patients	No. of included controls	Dynamics of biochemical markers	Effect measure, SMD	Quality evaluation, AMSTAR-2	Quality evaluation, GRADE
Pavlov et al. (2018), (22)	4 RCTs	254	256	ALT	−0.19 (95% CI − 0.38 to 0.00)	High	Moderate
				AST	−0.225 (95% CI: −0.45 to −0.001)		
				GGT	−0.47 (95% CI: −0.69 to −0.24)		
				ALP	−0.22 (95% CI: −0.45 to 0.002)		
Simental-Mendía M et al. (2020), (10)	22 RCTs	1,141	1,018	ALT	−0.16 (95% CI: −0.4 to −0.07)	Moderate	High
				AST	−0.18 (95% CI: −0.26 to −0.1)		
				GGT	−0.18 (95% CI: −0.27 to −0.1)		
				ALP	−0.24 (95%CI: −0.33 to −0.16)		
				Total Bilirubin	−0.09 (95% CI: −0.17 to −0.01)		
				Albumin	0.06 (95%CI:−0.03 to 0.14)		
Zhang et al. (2020) (45)	9 RCTs	403	497	ALT	−0.18 (95% CI -0.32 to −0.05)	High	High
				AST	−0.08 (95% CI -0.22 to 0.05)		
				GGT	−0.15 (95% CI -0.45 to 0.14)		
				ALP	−0.03 (95% CI -0.25 to 0.19)		
				Total Bilirubin	−0.02 (95% CI -0.16 to 0.21)		
				Albumin	0.05 (95% CI -0.16 to 0.25)		
Lin et al. (2022), (23)	8 RCTs	335	351	ALT	−0.20 (95%CI: −0.36 to −0.05)	High	High
				AST	−0.05 (95% CI: −0.2 to 0.11), *p* = 0.56		
				GGT	−0.54 (95%CI: −0.75 to −0.33)		
Patel et al. (2024), (24)	7 RCTs	379	381	ALT	−1.05 (95% CI –1.22 to −0.89)	Moderate	Moderate
				AST	−0.26 (95% CI: −0.41 to −0.11)		
				GGT	−0.66 (95% CI: −0.83 to −0.48)		

### 3.2 ROBIS assessment

Supplementary Material 1 provides a summary of the risk of bias evaluation conducted using the ROBIS tool. The greatest potential for bias was detected within the *Study eligibility criteria* domain. In contrast, the *Identification and selection of studies* domains showed the lowest risk. A similar pattern was observed for the assessment of Synthesis and findings. The ROBIS table (Supplementary Figure 2) is presented in Supplementary File 1.

### 3.3 GROOVE analysis

A total of 33 individual studies involving 5,015 participants were identified across all included systematic reviews and meta-analyses. For UDCA treatment in MASLD, the corrected covered area (CCA) was 12.28%, indicating high level of overlap; adjustment for chronological structural missingness yielded comparable values. Graphical representations of the GROOVE outcomes for the analysis is provided in Supplementary File 1.

### 3.4 Effectiveness of UDCA therapy

In this meta-analysis, the therapeutic effects of UDCA on biochemical markers associated with MASLD were evaluated across the available evidence. The pooled findings demonstrate that UDCA exerts measurable improvements on indicators of hepatocellular injury and intrahepatic cholestasis.

**Figure 2:**
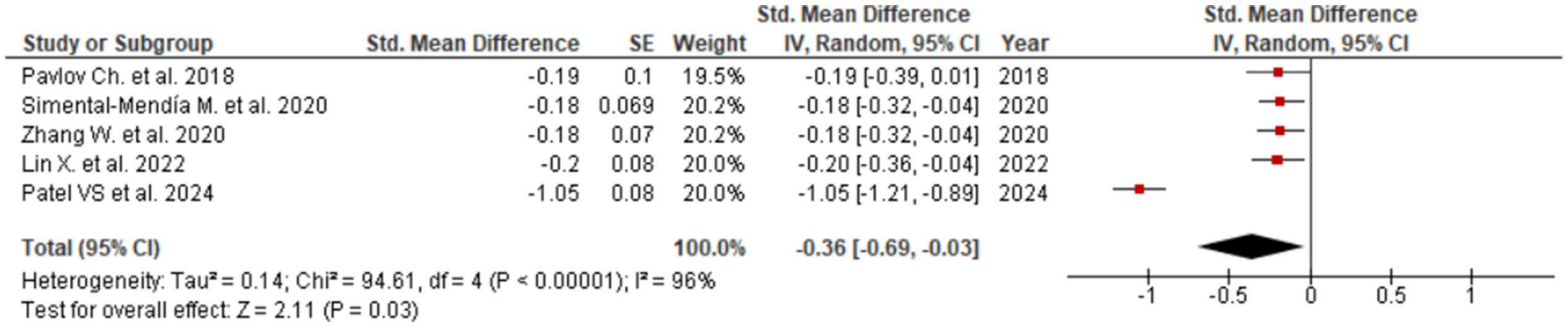
Evaluation of UDCA effect on ALT.

**Figure 3:**
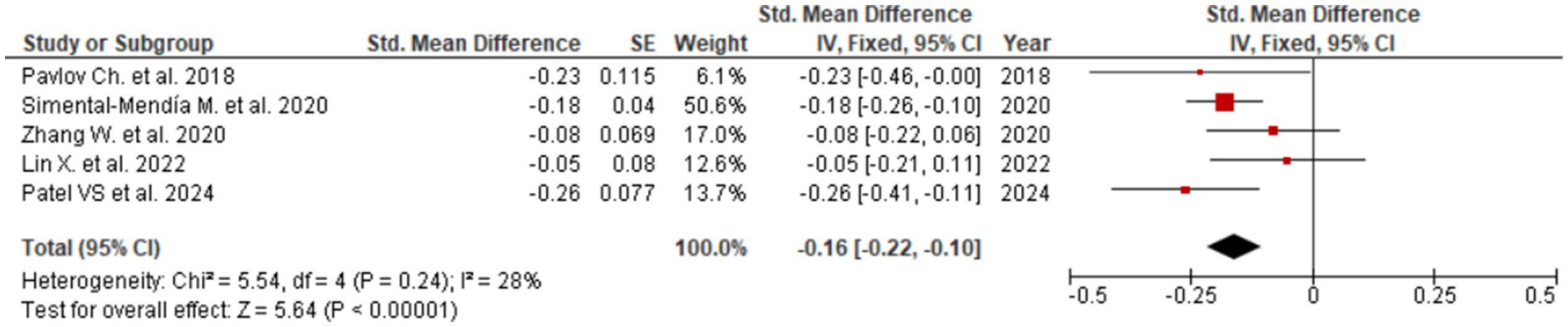
Evaluation of UDCA effect on AST.

Among all assessed biomarkers, the most pronounced effect was observed for ALT (SMD = −0.36; 95% CI: −0.69 to −0.03; Figure 2), indicating a meaningful reduction in hepatocellular injury and inflammatory activity. The corresponding forest plot shows a consistent direction of effect estimates across the included meta-analyses, with the majority favoring UDCA over control despite variability in effect size magnitude. A significant improvement was also identified for AST (SMD = −0.16; 95% CI: −0.22 to −0.10; Figure 3), supporting the interpretation that UDCA may attenuate ongoing hepatocyte damage characteristic of MASLD. Visual inspection of the forest plot demonstrates limited overlap of confidence intervals with the null effect and a generally homogeneous direction of effects.

**Figure 4:**
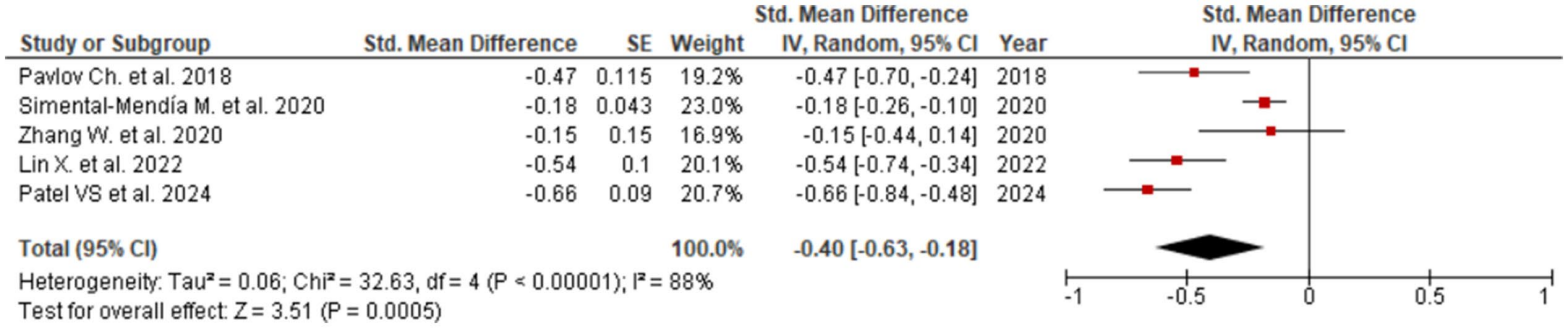
Evaluation of UDCA effect on GGT.

**Figure 5:**
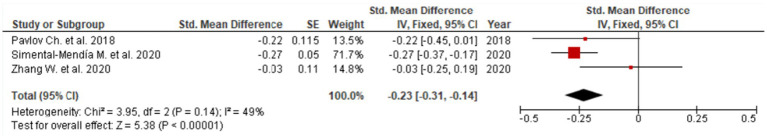
Evaluation of UDCA effect on ALP.

**Figure 6:**
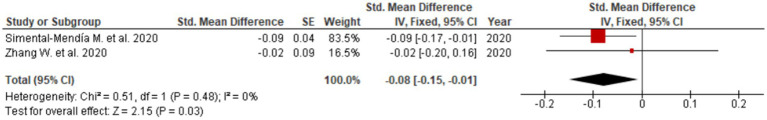
Evaluation of UDCA effect on total bilirubin.

Consistent reductions were similarly noted for GGT (SMD = −0.40; 95% CI: −0.63 to −0.18; Figure 4), reflecting beneficial effects on bile flow dynamics and resolution of intrahepatic cholestasis. The forest plot for GGT reveals a clear leftward shift of effect estimates across studies, indicating a robust and consistent cholestasis-modifying effect of UDCA. Statistically significant improvements were also observed for ALP (SMD = −0.23; 95% CI: −0.31 to −0.14; three studies, Figure 5) and total bilirubin (SMD = −0.08; 95% CI: −0.15 to −0.01; two studies, Figure 6). Although the direction of effect consistently favored UDCA, the relatively small number of studies contributing to these analyses warrants cautious interpretation.

**Figure 7:**
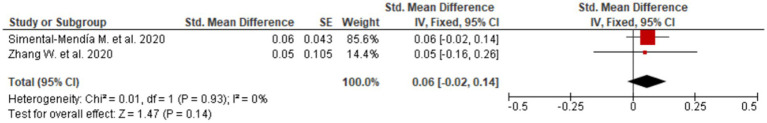
Evaluation of UDCA effect on albumin.

In contrast, albumin, a marker of hepatic synthetic function, showed minimal and non-significant change (SMD = 0.07; 95% CI: −0.02 to 0.15; two studies, Figure 7). The forest plot demonstrates effect estimates clustered around the null value with confidence intervals crossing zero, indicating a lack of clinically meaningful effect. This finding is consistent with the predominantly non-cirrhotic MASLD populations included in the analyzed studies, in whom albumin levels are typically preserved.

Publication bias was assessed using funnel plot asymmetry and Egger’s regression test. No evidence of significant publication bias was detected for ALT (Egger’s test *p* = 0.80), AST (*p* = 0.88), GGT (*p* = 0.27), or ALP (*p* = 0.44). However, Egger’s test indicated statistically significant asymmetry for total bilirubin (*p* = 0.0001) and albumin (*p* < 0.0001). These findings should be interpreted with extreme caution, as both analyses were based on a limited number of studies. Overall, visual inspection of funnel plots did not reveal marked asymmetry for the primary outcomes, suggesting a low likelihood of substantial publication bias affecting the main results.

### 3.5 Meta-regression analysis

**Figure 8:**
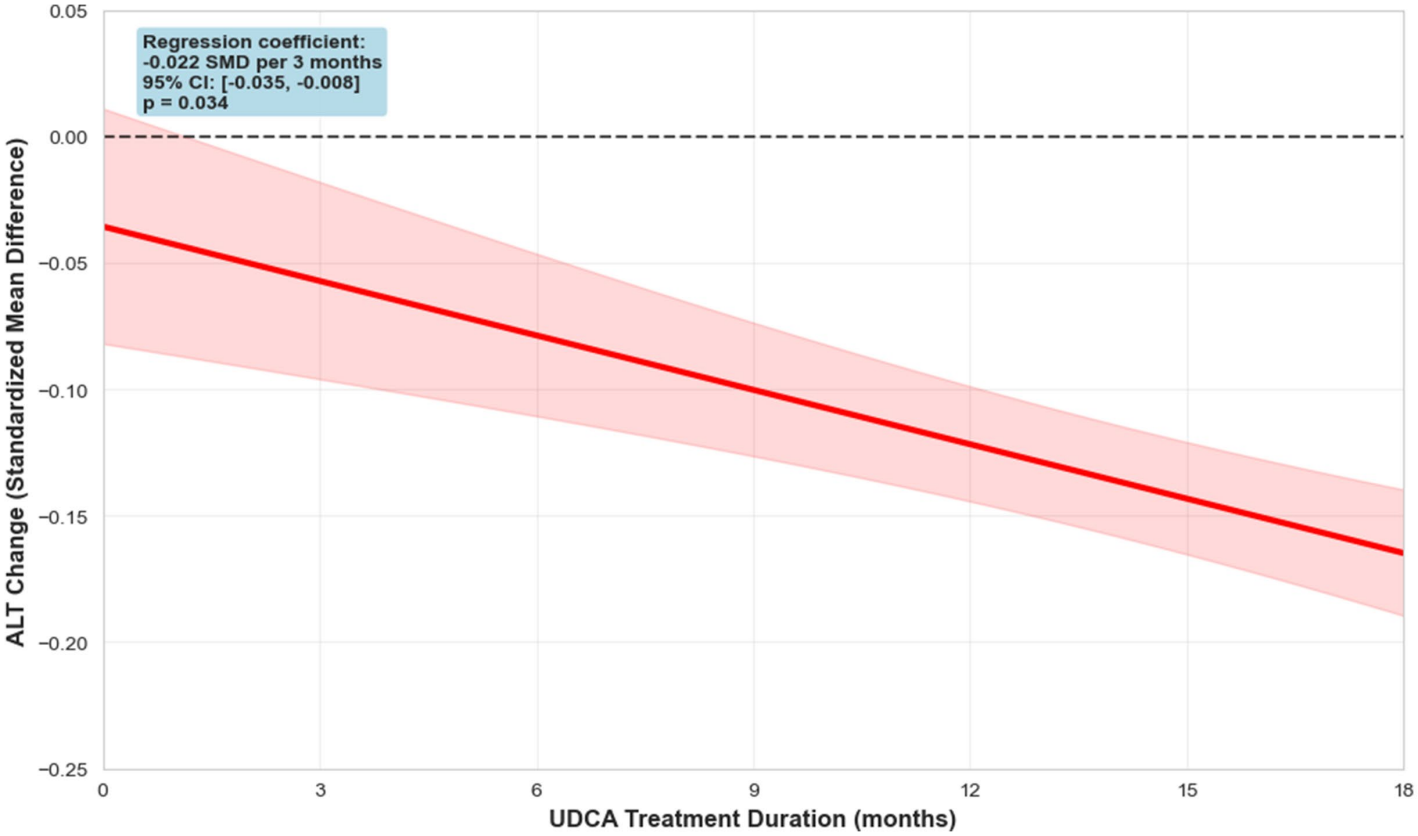
Meta-regression of ALT dynamics.

To further investigate the impact of treatment duration on therapeutic outcomes, we conducted a meta-regression analysis examining the relationship between study length and changes in ALT. Each additional month of therapy was associated with a significant reduction in ALT levels (−0.0084 SMD/month, 95% CI: −0.014 to −0.0030, *p*-value: 0.0149), with the most pronounced decrease observed at 6-month intervals. The analysis revealed a trend indicating that longer treatment periods were linked to greater ALT reductions, with the effect reaching statistical significance (SMD -0.02 per 3 months (Figure 8), 95% CI: −0.04 to −0.01, *p* = 0.015; per 6 months: -0.04 SMD, 95% CI: −0.07 to −0.02). These findings provide insight into the temporal dynamics of UDCA efficacy and emphasize the potential importance of treatment duration in optimizing biochemical responses in MASLD.

### 3.6 Subgroup analysis

To assess the robustness of our findings, we conducted a separate analysis including only studies rated as high quality according to the AMSTAR-2 scale. The results were as follows: ALT −0.19 (95% CI: −0.28 to −0.10), AST − 0.10 (95% CI: −0.19 to 0.00), and GGT − 0.41 (95% CI: −0.62 to −0.20). Notably, these values remain consistent with those observed in the overall sample, confirming the reliability of our primary analysis and supporting the conclusion that the observed effects are stable across studies of varying quality.

## 4 Discussion

### 4.1 Main findings

**Figure 9:**
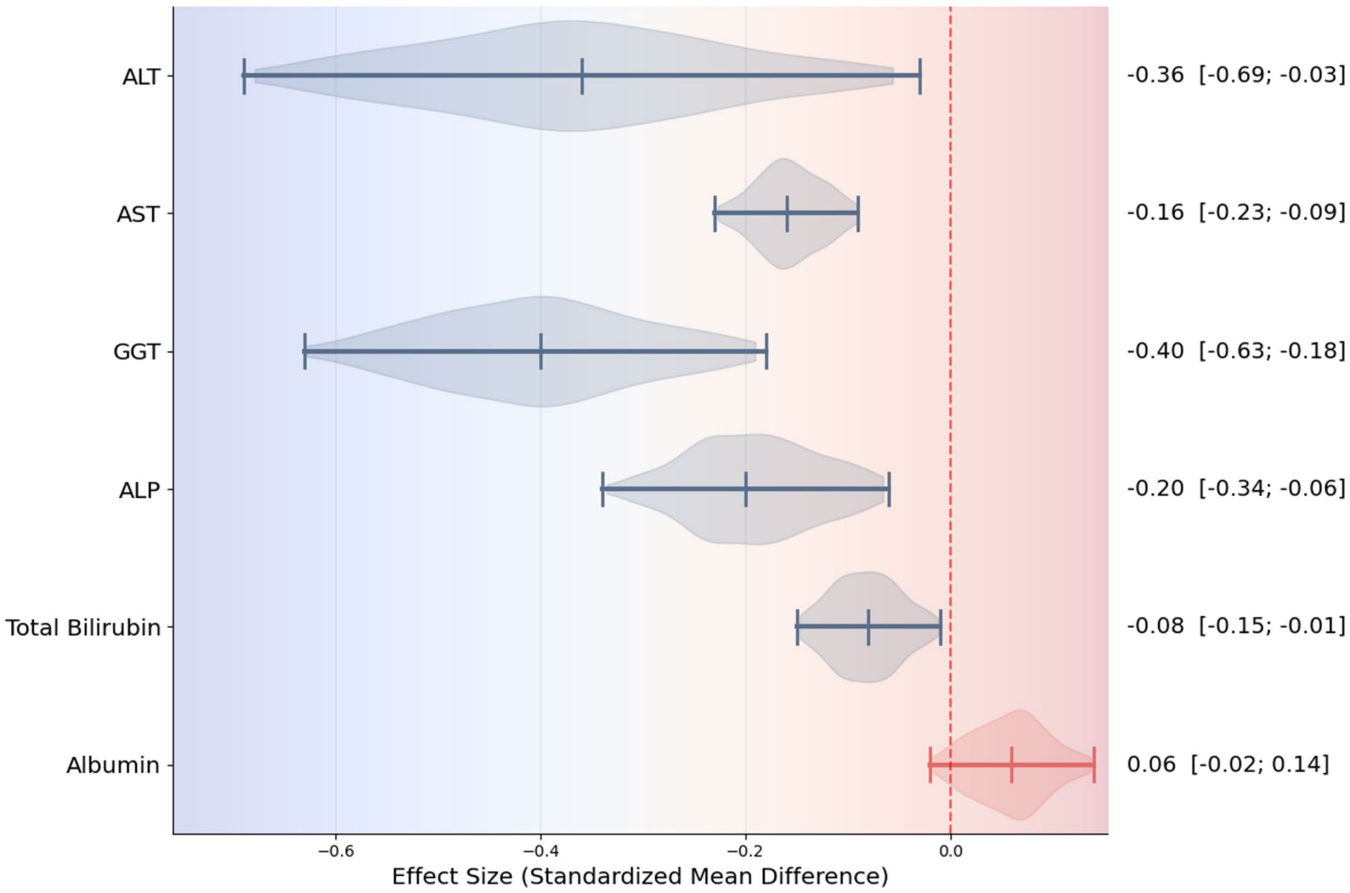
Violin plot summarizing effect sizes across biochemical markers.

In this umbrella review and meta-analysis, we systematically evaluated the therapeutic effects of UDCA on biochemical markers associated with MASLD. The results consistently demonstrate that UDCA provides significant improvements in markers of hepatocellular injury (ALT, AST) and intrahepatic cholestasis (GGT, ALP, total bilirubin). Among these, ALT and GGT exhibited the most pronounced reduction, suggesting a meaningful attenuation of hepatocellular inflammation and injury. These findings highlight previous observations from individual RCTs and meta-analyses reporting the hepatoprotective effects of UDCA (9–11, 22, 23). In addition, a violin plot summarizing the distribution of effect sizes across the included studies was constructed and is presented in Figure 9, providing a visual overview of data variability and central tendency.

Our analysis also revealed that UDCA exerts statistically significant effects on AST and ALP, which are widely recognized as secondary indicators of hepatocyte damage and bile duct function, respectively (25–27). The reductions in total bilirubin, although smaller in magnitude, further support UDCA’s potential to modulate cholestatic processes and improve bile acid homeostasis (28). Importantly, albumin levels showed a trend increase, although without statistical significance, consistent with the fact that our included populations primarily consisted of non-cirrhotic MASLD patients, in whom hepatic synthetic function is typically preserved (29, 30). This observation aligns with prior evidence indicating that UDCA’s benefits are primarily hepatoprotective and anti-inflammatory, rather than directly enhancing synthetic function in early-stage disease (31).

Our meta-regression analysis highlighted the temporal dimension of UDCA efficacy, revealing a significant association between longer treatment duration and greater reductions in ALT (−0.04, 95% CI: −0.07 to −0.02, *p* = 0.034). These findings suggest that sustained administration of UDCA may optimize hepatocellular outcomes and support its consideration in long-term management strategies for MASLD.

### 4.2 Potential mechanisms of therapeutic action of UDCA

The potential mechanisms underlying UDCA’s beneficial effects in MASLD are multifactorial. UDCA is known to stabilize hepatocyte membranes, reduce oxidative stress, inhibit apoptosis, and modulate inflammatory signaling pathways (32, 33). It also improves bile flow and promotes detoxification of hydrophobic bile acids, which may mitigate cholestasis-induced liver injury (34).

Recent studies further expand this mechanistic framework. UDCA has been shown to inhibit hepatocyte apoptosis through modulation of nuclear receptors, particularly via suppression of Farnesoid X Receptor signaling, as demonstrated in hemorrhagic shock models (35). Although FXR activation is generally hepatoprotective, its excessive stimulation under stress conditions can promote apoptosis, and UDCA’s ability to counterbalance this response adds another layer to its cytoprotective profile. Additionally, UDCA interacts with metabolic regulatory pathways such as the CYP4A14–PPARα axis, which has emerged as an important target in diet-induced MASLD (36). By normalizing CYP4A14 expression and enhancing PPARα activity, UDCA contributes to improved fatty acid oxidation, reduced lipid accumulation, and attenuation of steatohepatitis features.

Another significant mechanism involves UDCA-mediated regulation of autophagy and energy homeostasis. Studies indicate that UDCA activates AMP-activated protein kinase (AMPK), thereby restoring autophagic flux and reducing hepatocyte apoptosis—processes that are often impaired in MASLD (37). UDCA has also shown strong antioxidant and anti-apoptotic properties in models of hydrogen peroxide-induced hepatocyte injury, further supporting its role in maintaining mitochondrial integrity and limiting oxidative damage (38).

Furthermore, the improvement of autophagy during UDCA therapy may have direct relevance for steatosis regression in MASLD. Impaired autophagic clearance of lipid droplets (lipophagy) is considered a key mechanism underlying intrahepatic fat accumulation and steatosis progression (39). Activation of AMPK-dependent pathways by UDCA promotes restoration of autophagic flux, enhances fatty acid *β*-oxidation, and reduces intracellular lipid accumulation (40). Therefore, the observed biochemical improvements may reflect not only attenuation of inflammation and cholestasis but also a potential reduction of steatosis at the cellular level. Although direct histological evidence of steatosis regression with UDCA therapy remains limited, data on autophagy restoration provide a biologically plausible mechanism for possible structural liver improvement during prolonged treatment (37).

In addition, growing evidence highlights the connection between UDCA and the gut–liver axis. UDCA may beneficially modulate gut microbiome composition, bile acid pool hydrophobicity, and microbial metabolite profiles, collectively improving intestinal barrier function and reducing endotoxin-driven hepatic inflammation (41). These microbiome-mediated effects add an extra-hepatic dimension to UDCA’s therapeutic potential, suggesting that its benefits in MASLD extend beyond direct hepatocyte protection to systemic metabolic and immunological regulation.

Emerging evidence suggests that UDCA may exert favorable effects on metabolic and cardiometabolic parameters, including lipid metabolism, insulin sensitivity, and systemic inflammation, which are closely linked to MASLD progression (42). Meta-analytic data support these observations: UDCA therapy is associated with modest but statistically significant improvements in several glycemic markers. In one comprehensive meta-analysis, UDCA reduced fasting glucose levels (SMD = −0.17; 95% CI: −0.33 to −0.01), HbA1c concentrations (SMD = −0.16; 95% CI: −0.32 to −0.04), and plasma insulin levels (SMD = −0.18; 95% CI: −0.34 to −0.02), indicating enhanced insulin sensitivity across cohorts with NAFLD and NASH (13). More recent evidence further demonstrates that UDCA may exert favorable cardiometabolic modulation beyond glucose homeostasis (12). This meta-analysis reported a slight reduction in BMI (SMD = −0.056; 95% CI: −0.113 to 0.001) and a decrease in diastolic blood pressure (SMD = −0.08; 95% CI: −0.14 to −0.03).

Collectively, these findings reinforce the concept that UDCA exerts benefits through a constellation of hepatocellular, metabolic, and extrahepatic mechanisms, reflecting its unique position among available therapies for MASLD.

### 4.3 Strengths and limitations

Despite the encouraging biochemical improvements observed in our study, several limitations should be acknowledged. First, the included meta-analyses varied in terms of patient populations However, heterogeneity related to UDCA exposure was limited, as dosing regimens were highly consistent across the included randomized controlled trials, with most studies using standard therapeutic doses of 10–15 mg/kg/day. In addition, treatment duration was broadly comparable, typically ranging from 3 to 12 months, reducing the likelihood that variability in dose or duration substantially influenced the pooled results. Although we conducted subgroup and meta-regression analyses to account for residual differences (demonstrating a significant association between treatment duration and ALT reduction) residual confounding cannot be fully excluded. Second, our meta-analysis was constrained by the availability of data on key biomarkers of interest. As not every study reported the complete set of relevant parameters, the pooled estimates for some outcomes were derived from 2 to 3 studies, which may limit the statistical power and precision of our conclusions. Third, a substantial overlap of primary studies across the included meta-analyses was observed (CCA = 12.28%), indicating a high degree of shared evidence. This overlap was mainly attributable to a small number of frequently cited randomized controlled trials that were consistently included across multiple meta-analyses and did not significantly contribute to the estimates while performing leave-one-out sensitivity analysis. Fourth, our analysis assessed only biochemical parameters. We did not include data from imaging or histology that reflect actual changes in hepatic steatosis or fibrosis, as such studies with longitudinal assessment are currently scarce. For example, Nadinskaia et al. (8), Dudanova et al. (43), and Brekhunets et al. (44) reported improvements in non-invasive fibrosis/steatosis indices with UDCA therapy. Therefore, while our results indicate favorable biochemical changes, they cannot reliably demonstrate regression of steatosis or fibrosis at the tissue level.

Nonetheless, the present umbrella review offers a comprehensive synthesis of existing evidence, demonstrating that UDCA consistently reduces markers of hepatocellular injury and cholestasis in MASLD. The meta-regression findings suggest that treatment duration may further enhance these effects, providing guidance for optimizing therapeutic regimens. Taken together, these results support the potential role of UDCA as an adjunctive therapy in MASLD management. Future research should aim to integrate biochemical, histological, and clinical outcomes to determine the full scope of UDCA’s efficacy, and explore combination therapies targeting metabolic dysfunction, inflammation, and fibrosis.

## 5 Conclusion

In conclusion, UDCA demonstrates reproducible and clinically relevant effects on key biochemical markers of MASLD, with the strongest impact observed on ALT and GGT. The consistency of findings across studies of varying quality, coupled with the observed temporal relationship in meta-regression, underscores the therapeutic potential of UDCA and warrants further investigation in well-designed, long-term trials.

## Data Availability

The original contributions presented in the study are included in the article/Supplementary material, further inquiries can be directed to the corresponding authors.

## References

[1] Kim DH Ko D Danpanichkul P Ho GJK Tan FXN Sasikumar NA . Longitudinal clinical outcomes and mortality from steatotic liver disease: a meta-analysis. Am J Med. (2025) 138:1238–1247.e4. doi: 10.1016/j.amjmed.2025.04.027, 40316227

[2] Younossi ZM Kalligeros M Henry L. Epidemiology of metabolic dysfunction-associated steatotic liver disease. Clin Mol Hepatol. (2025) 31:S32–50. doi: 10.3350/cmh.2024.0431, 39159948 PMC11925440

[3] Teng ML Ng CH Huang DQ Chan KE Tan DJ Lim WH . Global incidence and prevalence of nonalcoholic fatty liver disease. Clin Mol Hepatol. (2023) 29:S32–42. doi: 10.3350/cmh.2022.0365, 36517002 PMC10029957

[4] Assimakopoulos K Karaivazoglou K Tsermpini EE Diamantopoulou G Triantos C. Quality of life in patients with nonalcoholic fatty liver disease: a systematic review. J Psychosom Res. (2018) 112:73–80. doi: 10.1016/j.jpsychores.2018.07.004, 30097139

[5] Stefan N Häring HU Cusi K. Non-alcoholic fatty liver disease: causes, diagnosis, cardiometabolic consequences, and treatment strategies. Lancet Diabetes Endocrinol. (2019) 7:313–24. doi: 10.1016/S2213-8587(18)30154-2, 30174213

[6] Godoy-Matos AF Silva Júnior WS Valerio CM. NAFLD as a continuum: from obesity to metabolic syndrome and diabetes. Diabetol Metab Syndr. (2020) 12:60. doi: 10.1186/s13098-020-00570-y 32684985 PMC7359287

[7] Tacke F Horn P Wai-Sun Wong V Ratziu V Bugianesi E Francque S . EASL–EASD–EASO clinical practice guidelines on the management of metabolic dysfunction-associated steatotic liver disease (MASLD). J Hepatol. (2024) 81:492–542. doi: 10.1016/j.jhep.2024.04.031 38851997

[8] Nadinskaia M Maevskaya M Ivashkin V Kodzoeva K Pirogova I Chesnokov E . Ursodeoxycholic acid as a means of preventing atherosclerosis, steatosis and liver fibrosis in patients with nonalcoholic fatty liver disease. World J Gastroenterol. (2021) 27:959–75. doi: 10.3748/wjg.v27.i10.959, 33776366 PMC7968130

[9] Xiang Z Chen Y Ma K Ye Y Zheng L Yang Y . The role of ursodeoxycholic acid in non-alcoholic steatohepatitis: a systematic review. BMC Gastroenterol. (2013) 13:140. doi: 10.1186/1471-230x-13-140, 24053454 PMC3848865

[10] Simental-Mendía M Sánchez-García A Simental-Mendía LE. Effect of ursodeoxycholic acid on liver markers: a systematic review and meta-analysis of randomized placebo-controlled clinical trials. Br J Clin Pharmacol. (2020) 86:1476–88. doi: 10.1111/bcp.14311, 32285958 PMC7373700

[11] Simental-Mendía LE Simental-Mendía M Sánchez-García A Banach M Serban MC Cicero AFG . Impact of ursodeoxycholic acid on circulating lipid concentrations: a systematic review and meta-analysis of randomized placebo-controlled trials. Lipids Health Dis. (2019) 18:88. doi: 10.1186/s12944-019-1041-4, 30954082 PMC6451779

[12] Rashidbeygi E Rasaei N Amini MR Salavatizadeh M Mohammadizadeh M Hekmatdoost A. The effects of ursodeoxycholic acid on cardiometabolic risk factors: a systematic review and meta-analysis of randomized controlled trials. BMC Cardiovasc Disord. (2025) 25:125. doi: 10.1186/s12872-025-04549-3, 39984850 PMC11844182

[13] Sánchez-García A Sahebkar A Simental-Mendía M Simental-Mendía LE. Effect of ursodeoxycholic acid on glycemic markers: a systematic review and meta-analysis of clinical trials. Pharmacol Res. (2018) 135:144–9. doi: 10.1016/j.phrs.2018.08.008, 30099154

[14] Newton KP Lavine JE Wilson L Behling C Vos MB Molleston JP . Alanine aminotransferase and gamma-glutamyl transpeptidase predict histologic improvement in pediatric nonalcoholic steatohepatitis. Hepatology. (2021) 73:937–51. doi: 10.1002/hep.31317, 32416645 PMC7669708

[15] Aromataris E Fernandez R Godfrey CM Holly C Khalil H Tungpunkom P. Summarizing systematic reviews. Int J Evid Based Healthc. (2015) 13:132–40. doi: 10.1097/XEB.0000000000000055, 26360830

[16] Ioannidis JPA. Integration of evidence from multiple meta-analyses: a primer on umbrella reviews, treatment networks and multiple treatments meta-analyses. Can Med Assoc J. (2009) 181:488–93. doi: 10.1503/cmaj.081086, 19654195 PMC2761440

[17] Page MJ McKenzie JE Bossuyt PM Boutron I Hoffmann TC Mulrow CD . The PRISMA 2020 statement: an updated guideline for reporting systematic reviews. BMJ. (2021):n71. doi: 10.1136/bmj.n71 33782057 PMC8005924

[18] Shea BJ Reeves BC Wells G Thuku M Hamel C Moran J . AMSTAR 2: a critical appraisal tool for systematic reviews that include randomised or non-randomised studies of healthcare interventions, or both. BMJ. (2017):j4008. doi: 10.1136/bmj.j4008 28935701 PMC5833365

[19] Schünemann HJ Cuello C Akl EA Mustafa RA Meerpohl JJ Thayer K . GRADE guidelines: 18. How ROBINS-I and other tools to assess risk of bias in nonrandomized studies should be used to rate the certainty of a body of evidence. J Clin Epidemiol. (2019) 111:105–14. doi: 10.1016/j.jclinepi.2018.01.012, 29432858 PMC6692166

[20] Brożek JL Akl EA Alonso-Coello P Lang D Jaeschke R Williams JW . Grading quality of evidence and strength of recommendations in clinical practice guidelines. Allergy. (2009) 64:19210357:669–77. doi: 10.1111/j.1398-9995.2009.01973.x 19210357

[21] Bracchiglione J Meza N Bangdiwala SI de Niño Guzmán E Urrútia G Bonfill X . Graphical representation of overlap for overviews: GROOVE tool. Res Synth Methods. (2022) 13:381–8. doi: 10.1002/jrsm.1557 35278030

[22] Pavlov СS Varganova DL Semenistaia MC Kuznetsova EA Usanova AA Svistunov AA. Ursodeoxycholic acid: efficacy and safety in the treatment of nonalcoholic fatty liver disease (Meta-analysis). Ann Russ Acad Med Sci. (2018) 73:294–305. doi: 10.15690/vramn975

[23] Lin X Mai M He T Huang H Zhang P Xia E . Efficiency of ursodeoxycholic acid for the treatment of nonalcoholic steatohepatitis: a systematic review and meta-analysis. Expert Rev Gastroenterol Hepatol. (2022) 16:537–45. doi: 10.1080/17474124.2022.2083605, 35617696

[24] Patel VS Mahmood SF Bhatt KH Khemkar RM Jariwala DR Harris B . Ursodeoxycholic acid\‘s effectiveness in the Management of Nonalcoholic Fatty Liver Disease: a systematic review and Meta-analysis. Euroasian J Hepatogastroenterol. (2024) 14:92–8. doi: 10.5005/jp-journals-10018-1434, 39022193 PMC11249908

[25] Cho EJ Jeong SM Chung GE Yoo JJ Cho Y Lee KN . Gamma-glutamyl transferase and risk of all-cause and disease-specific mortality: a nationwide cohort study. Sci Rep. (2023) 13:1751. doi: 10.1038/s41598-022-25970-0, 36720971 PMC9888340

[26] Kwo PY Cohen SM Lim JK. ACG clinical guideline: evaluation of abnormal liver chemistries. Am J Gastroenterol. (2017) 112:18–35. doi: 10.1038/ajg.2016.517, 27995906

[27] Iluz-Freundlich D Zhang M Uhanova J Minuk GY. The relative expression of hepatocellular and cholestatic liver enzymes in adult patients with liver disease. Ann Hepatol. (2020) 19:204–8. doi: 10.1016/j.aohep.2019.08.004, 31628070

[28] Cifuentes-Silva E Cabello-Verrugio C. Bile acids as signaling molecules: role of Ursodeoxycholic acid in Cholestatic liver disease. Curr Protein Pept Sci. (2024) 25:206–14. doi: 10.2174/1389203724666230818092800, 37594109

[29] Angulo P Kleiner DE Dam-Larsen S Adams LA Bjornsson ES Charatcharoenwitthaya P . Liver fibrosis, but no other histologic features, is associated with Long-term outcomes of patients with nonalcoholic fatty liver disease. Gastroenterology. (2015) 149:389–397.e10. doi: 10.1053/j.gastro.2015.04.043, 25935633 PMC4516664

[30] Xiao G Zhu S Xiao X Yan L Yang J Wu G. Comparison of laboratory tests, ultrasound, or magnetic resonance elastography to detect fibrosis in patients with nonalcoholic fatty liver disease: a meta-analysis. Hepatology. (2017) 66:1486–501. doi: 10.1002/hep.29302, 28586172

[31] Beuers U. Drug insight: mechanisms and sites of action of ursodeoxycholic acid in cholestasis. Nat Clin Pract Gastroenterol Hepatol. (2006) 3:318–28. doi: 10.1038/ncpgasthep0521, 16741551

[32] Roma MG Toledo FD Boaglio AC Basiglio CL Crocenzi FA Sánchez Pozzi EJ. Ursodeoxycholic acid in cholestasis: linking action mechanisms to therapeutic applications. Clin Sci. (2011) 121:523–44. doi: 10.1042/CS20110184, 21854363

[33] Lazaridis KN Gores GJ Lindor KD. Ursodeoxycholic acid ‘mechanisms of action and clinical use in hepatobiliary disorders. J Hepatol. (2001) 35:134–46. doi: 10.1016/s0168-8278(01)00092-7, 11495032

[34] Stan SI Biciușcă V Clenciu D Mitrea A Boldeanu MV Durand P . The therapeutic mechanisms and beneficial effects of ursodeoxycholic acid in the treatment of nonalcoholic fatty liver disease: a systematic review. Med Pharm Rep. (2023) 97:12–25. doi: 10.15386/mpr-2629, 38344336 PMC10852123

[35] Wang L Rui X He HW Zhou X Long Y. Ursodeoxycholic acid (UDCA) reduces hepatocyte apoptosis by inhibiting Farnesoid X receptor (FXR) in hemorrhagic shock (HS). Curr Mol Med. (2023) 23:550–8. doi: 10.2174/1566524022666220525152811, 35619282

[36] Li B Zhong P Zhang X Li C Luan M Chen Y . CYP4A14-PPARα axis serves as a therapeutic target for ursodeoxycholic acid in ameliorating high-fat diet-induced MASLD. J Nutr Biochem. (2026) 147:110138. doi: 10.1016/j.jnutbio.2025.110138, 41061809

[37] Wu P Zhao J Guo Y Yu Y Wu X Xiao H. Ursodeoxycholic acid alleviates nonalcoholic fatty liver disease by inhibiting apoptosis and improving autophagy via activating AMPK. Biochem Biophys Res Commun. (2020) 529:834–8. doi: 10.1016/j.bbrc.2020.05.128, 32595039

[38] Wang X Liang G Zhou Y Ni B Zhou X. Ameliorative effect and mechanism of ursodeoxycholic acid on hydrogen peroxide-induced hepatocyte injury. Sci Rep. (2024) 14:4446. doi: 10.1038/s41598-024-55043-3, 38395998 PMC10891090

[39] Hwang JS Lai TH Kim DR. Targeting lipophagy in liver diseases: impact on oxidative stress and steatohepatitis. Antioxidants. (2025) 14:908. doi: 10.3390/antiox14080908, 40867807 PMC12382931

[40] Fang C Pan J Qu N Lei Y Han J Zhang J . The AMPK pathway in fatty liver disease. Front Physiol. (2022) 13:970292. doi: 10.3389/fphys.2022.970292, 36203933 PMC9531345

[41] Mao Q Lin B Zhang W Zhang Y Zhang Y Cao Q . Understanding the role of ursodeoxycholic acid and gut microbiome in non-alcoholic fatty liver disease: current evidence and perspectives. Front Pharmacol. (2024) 15:1371574. doi: 10.3389/fphar.2024.1371574 38576492 PMC10991717

[42] Lakić B Škrbić R Uletilović S Mandić-Kovačević N Grabež M Šarić MP . Beneficial effects of ursodeoxycholic acid on metabolic parameters and oxidative stress in patients with type 2 diabetes mellitus: a randomized double-blind, placebo-controlled clinical study. J Diabetes Res. (2024) 2024:4187796. doi: 10.1155/2024/4187796 PMC10919985 38455850

[43] Dudanova OP Shipovskaya AA Larina NA Kurbatova IV Radchenko VG Seliverstov PV. Hepatotropic and metabolic properties of ursodeoxycholic acid for nonalcocholic liver disease. Exp Clin Gastroenterol. (2024) 4:4–9. doi: 10.31146/1682-8658-ecg-224-4-4-9

[44] Brekhunets RM Dicheva DT Khurmatullina AR Andreev DA Kulieva АК Berezutskaya ОЕ . Efficacy of combination therapy with lifestyle modification and ursodeoxycholic acid in patients with non-alcoholic fatty liver disease: results of a prospective study. Med Sov. (2025) 19:75–83. doi: 10.21518/ms2025-563

[45] Zhang W Tang Y Huang J Hu H. Efficacy of ursodeoxycholic acid in nonalcoholic fatty liver disease: An updated meta-analysis of randomized controlled trials. Asia Pac J Clin Nutr. (2020) 29:696–705. doi: 10.6133/apjcn.202012_29(4).0004, 33377363

