# Supplementary material for: Efficacy of ursodeoxycholic acid in metabolic dysfunction-associated steatotic liver disease: an umbrella review of meta-analyses on liver enzymes
Source: Front Med (Lausanne). 2026 Feb 13;13:1771830. doi: 10.3389/fmed.2026.1771830 (PMC12945775; doi:10.3389/fmed.2026.1771830)
Supplement: Supplementary file 1 [file Supplementary_file_1.docx]

Figure 2. ROBIS assessment of the studies.


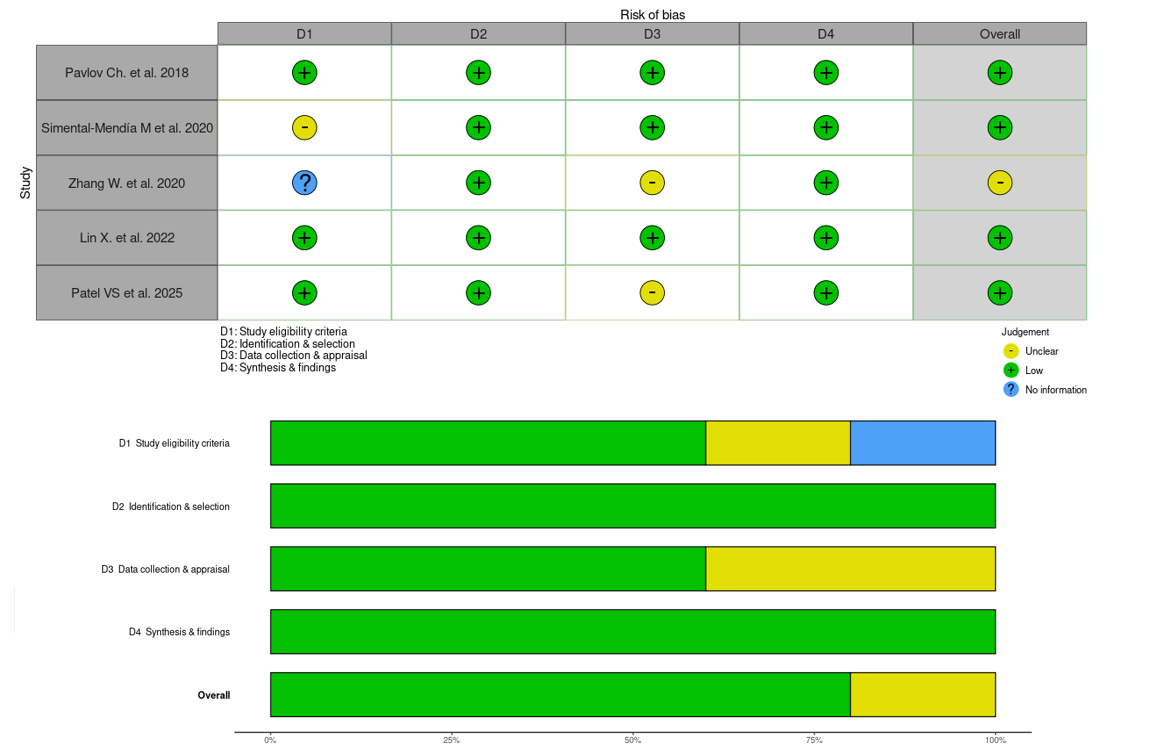


Figure 3. GROOVE Analysis


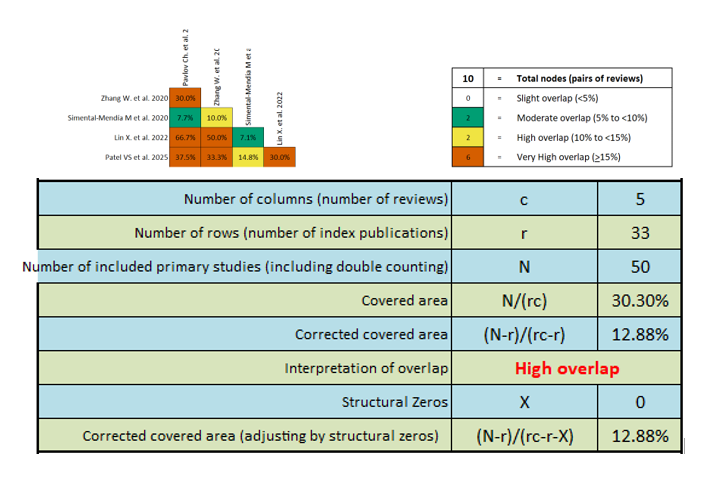


**Full Search Strategy and Records Retrieved**

1. **EMBASE** (n = 51)
   ('fatty liver'/exp OR 'nonalcoholic fatty liver disease'/exp OR NAFLD:ti,ab OR MASLD:ti,ab OR NASH:ti,ab) AND ('ursodeoxycholic acid'/exp OR UDCA:ti,ab) AND ('systematic review':ti,ab OR 'meta-analysis':ti,ab)
2. **MEDLINE/PubMed** (n = 18)
   ("Fatty Liver"[MeSH] OR "Non-alcoholic Fatty Liver Disease" OR NAFLD OR MASLD OR NASH "metabolic dysfunction–associated steatotic liver disease") AND ("Ursodeoxycholic Acid"[MeSH] OR UDCA OR ursodiol)
3. **Cochrane Library** (n = 3)
   ("fatty liver" OR "non-alcoholic fatty liver disease" OR NAFLD OR MASLD OR NASH OR "metabolic dysfunction–associated steatotic liver disease")
   AND
   ("ursodeoxycholic acid" OR UDCA OR ursodiol)
4. **Scopus** (n = 240)
   TITLE-ABS-KEY (NAFLD OR NASH OR MASLD) AND TITLE-ABS-KEY ( "ursodeoxycholic acid") AND (LIMIT-TO (DOCTYPE, "re"))
